# Observing the Testing Effect using *Coursera* Video-Recorded Lectures: A Preliminary Study

**DOI:** 10.3389/fpsyg.2015.02064

**Published:** 2016-01-29

**Authors:** Paul Zhihao Yong, Stephen Wee Hun Lim

**Affiliations:** Department of Psychology, Faculty of Arts and Social Sciences, National University of SingaporeSingapore, Singapore

**Keywords:** retrieval-based learning, the testing effect, video-based learning, experimental education, educational psychology

## Abstract

We investigated the testing effect in *Coursera* video-based learning. One hundred and twenty-three participants either (a) studied an instructional video-recorded lecture four times, (b) studied the lecture three times and took one recall test, or (c) studied the lecture once and took three tests. They then took a final recall test, either immediately or a week later, through which their learning was assessed. Whereas repeated studying produced better recall performance than did repeated testing when the final test was administered immediately, testing produced better performance when the final test was delayed until a week after. The testing effect was observed using *Coursera* lectures. Future directions are documented.

## Introduction

In the last three decades, we witnessed a rapid expansion of testing in the vast majority of countries worldwide. Students are exposed to tests which are, more so than before, standardized and associated with high-stake outcomes. These tests take various formats (comprising of, e.g., multiple-choice or short-answer questions) and are used to assess students' knowledge, understanding, and aptitude, as well as to summarize performance, assign grades, and rank students. Yet, many educators believe that testing is overly emphasized. The view is that testing should be minimized, so that time will not be taken away from, but can be devoted toward, classroom learning and instruction. Moreover, testing can create high anxieties among students (Wittmaier, 1972) or burdens for educators who would have to grade papers (see Roediger and Karpicke, 2006b), thus both parties tend to view tests negatively.

Such views are disheartening because testing, when used judiciously, can actually conduce learning benefits. Intuitively, if teachers were to administer tests on a regular basis, students would have distributed their studying throughout the academic semester, instead of cramming prior to final exams (Bangert-Drowns et al., 1991; Leeming, 2002). In fact, there is a solid body of research which demonstrates that testing promotes effective long-term learning. This phenomenon is known as the testing effect (e.g., Carrier and Pashler, 1992; Wheeler and Roediger, 1992; Chan et al., 2006), which has also been called test-enhanced learning (Roediger and Karpicke, 2006a) or, more recently, retrieval-based learning (Karpicke, 2012).

## Retrieval-based learning

Traditionally, learning has been associated with the encoding of new knowledge and experiences whilst retrieval serves only as a means for assessing learning. Under this view, increasing opportunities for encoding or study events would promote learning, whereas increasing opportunities for retrieval would not, to the extent that retrieval were regarded merely as an assessment of a person's knowledge. Yet, retrieval processes can impact learning in diverse ways. Specifically, there are indirect and direct effects of retrieval on learning (Roediger and Karpicke, 2006b). An indirect effect of retrieval obtains when retrieval enhances learning by virtue of some secondary mediating process. For example, when one attempts to retrieve knowledge, the outcome of that attempt would constitute feedback for the learner which would, in turn, guide him or her to (re)deploy study time or change encoding strategies (Pyc and Rawson, 2010). Retrieval also produces direct effects on learning, since engaging in the process of retrieval itself produces learning. This is because every time we retrieve knowledge, that knowledge is modified, and the ability to reconstruct that knowledge during future instances is enhanced.

In the standard retrieval-based learning paradigm, learners either studied educational materials repeatedly, or studied and then practiced retrieving the materials, before taking a final test through which their learning was assessed. In Roediger and Karpicke (2006a; Experiment 2), students either studied a prose passage once and took three free recall tests about the material, studied the passage three times and took one test, or basically studied the passage four times. They then took a final retention test either 5 min or a week later. Massed studying has been known to produce short-term knowledge retention benefits (see Balota et al., 1989). Accordingly, Roediger and Karpicke (2006a) found that students who studied the material repeatedly performed better when the retention test was administered immediately. The crucial observation, however, was that students who practiced retrieving performed better than did students who merely studied when the test was administered a week later, demonstrating the benefits of retrieval practice on longer-term retention of educationally relevant knowledge (see, also, Lim et al., 2015).

## The present study

Online learning platforms, of which video-recorded lectures are a central feature, are becoming popular today. As a result, learners enjoy access to a wide range of learning resources and much flexibility to learn at their own preferred pace. But, the success of video-based learning, more so than does that of traditional classroom learning, hinges heavily on independent learning and, thereby, the learners' sole responsibility to stay on track. To these ends, there is burgeoning interest toward ways of enhancing such video-based learning (see Schacter and Szpunar, 2015, for a discussion).

Our goal was first to provide a conceptual replication (see Bohannon, 2015; Open Science Collaboration, 2015 concerning the importance of replicability of psychological science) of the benefits of testing in, specifically, *Coursera* video-based learning (see also, Butler and Roediger, 2007; Johnson and Mayer, 2009; Szpunar et al., 2013, 2014). We particularly aimed, in this preliminary study, to provide clear, solid data in support of a larger project in our Lab, via which we hope to eventually assist actual *Coursera* learners to learn more effectively through the use of tests. The rationale for centering on *Coursera*, an educational technology company that offers MOOCs (massive open online courses), was motivated by its fast-growing prominence in the world of online learning. Within months following its establishment in January 2012, it reached more than 1.7 million learners, developing—in the words of *Coursera*'s Co-Founder Andrew Ng—“faster than Facebook” (Pappano, 2012).

We tested two hypotheses. First, repeated studying—relative to repeated testing—would improve *Coursera* video-based learning performance, when the final test was administered immediately. Second, and contrastingly, testing would produce better performance when the final test was administered a week later.

## Methods

### Participants

One hundred and twenty-three students from the National University of Singapore, aged 18–26, participated either voluntarily, to fulfill course requirements, or whilst receiving monetary compensation ($10 for an hour of participation). All participants reported normal or corrected-to-normal vision, with no history of hearing impairment. This research was conducted with the appropriate ethics review board approval by the National University of Singapore, and participants have granted their written informed consent.

### Materials

Two *Coursera* lectures were used. Each lecture covered a single topic—“Music History” or “Brain Matter.” The “Music History” and “Brain Matter” videos spanned 2 min 40 s and 2 min 52 s, respectively. Both videos were transcribed into text passages comprising of 496 and 465 words, respectively, and each video was divided into 30 unit idea units for scoring purposes (see Supplementary Material section for examples). In both videos, the respective lecturers remained visible to the viewers. Participants were randomly assigned to view one of the two lectures; 62 participants watched “Music History” whereas 61 participants watched “Brain Matter.”

### Design

The experiment used a 3 × 2 fully between-subjects design. The two independent variables were (1) learning condition: (a) repeated study (SSSS), (b) study with a single free recall test (SSST), and (c) study with repeated testing (STTT), and (2) retention interval: (a) 5-min vs. (b) 1-week retention interval. The dependent variable was the mean proportion of idea units recalled.

### Procedure

Participants underwent two phases. Phase 1 comprised of four consecutive periods. Participants were first briefed on what they would experience during the four consecutive periods. Participants in the SSSS condition studied the video lecture for four 6-min periods. During each study period, participants were instructed to view the lecture once through and, thereafter, return to study parts which they found to be more difficult. Participants in the SSST condition studied the lecture in the same way as did those in the SSSS condition, but for three 6-min study periods; they then took one recall test during the fourth 6-min period. Participants in the STTT condition studied the lecture for one 6-min study period and then took three consecutive recall tests during the next three 6-min periods. Participants in the SSST and STTT conditions were instructed to recall as much of the lecture material as they could during each recall period.

At the end of Phase 1, participants were given an interim questionnaire. Participants were asked to indicate on a 7-point Likert scale: (1) how interesting they thought the online lecture was (1 = “very boring”; 7 = “very interesting”), (2) how understandable the content of the online lecture was (1 = “very difficult to understand”; 7 = “very easy to understand”), (3) how understandable the accent of the lecturer was (1 = “very difficult to understand”; 7 = “very easy to understand”), (4) how well they thought they would remember the online lecture in 5 min' or in a week's time, depending on which condition the participants were in (1 = “not very well”; 7 = “very well”), (5) whether they have watched the online lecture before (“yes” or “no”) and (6) how well they knew the subject matter covered prior to viewing the online lecture (1 = “not very well”; 7 = “very well”). Participants then completed a filler task comprising of math multiplication problems which lasted 5 min.

Phase 2 ensued after either a 5-min or a 1-week retention interval. Participants in the 5-min retention interval conditions stayed on for the experiment immediately after completing Phase 1, whereas participants in the 1-week retention interval conditions left after Phase 1 and returned exactly a week later. During Phase 2, participants were instructed to freely recall the lecture material which they had previously studied in Phase 1; the recall instructions were identical to those given during retrieval periods of Phase 1. The final recall test lasted 10 min.

## Results

Participants' recall responses were scored by awarding 1 point for each correctly recalled idea unit; the maximum score was 30. Twelve sets of recall tests were scored by two independent raters, and the Pearson product-moment correlation (*r*) between their scores was 0.99. Given the high inter-rater reliability, the remaining test sets were scored by a single rater (this procedure was identical to that implemented by Roediger and Karpicke, 2006a).

The mean ratings of the questionnaire items appear in Table 1. Importantly, all participants reported that they had not, prior to the experiment, come across the video lecture on which they were tested. For the 5-min retention interval group, participants in the SSSS condition seemed more confident that they would remember the online lecture material (*M* = 5.76) than seemed participants in the SSST (*M* = 5.45) or STTT (*M* = 5.00) condition. For the 1-week retention interval group, participants in the SSSS condition also seemed more confident that they would remember the online lecture material (*M* = 4.35) than seemed participants in the SSST (*M* = 4.16) or STTT (*M* = 3.84) condition (see, also, Roediger and Karpicke, 2006a).

**Table 1:** **Mean ratings of the questionnaire items**.

**Learning condition**	**Rating**					
	**Interesting**	**Understandable**	**Accent**	**Remember**	**Watched?**	**Subject matter**
SSSS, 5-min	4.81	6.29	6.48	5.76	21 N	3.09
SSST, 5-min	5.36	6.81	6.45	5.45	22 N	2.82
STTT, 5-min	4.95	6.00	6.14	5.00	22 N	3.18
SSSS, 1-week	4.75	6.65	6.65	4.35	20 N	2.35
SSST, 1-week	5.32	6.58	6.26	4.16	19 N	3.05
STTT, 1-week	5.21	6.37	6.37	3.84	19 N	2.79

*S denotes study, whereas T denotes test. Labels for “Watched?”: N denotes “No” (did not watch). Participants rated on a 7-point Likert scale*.

**Figure 1:**
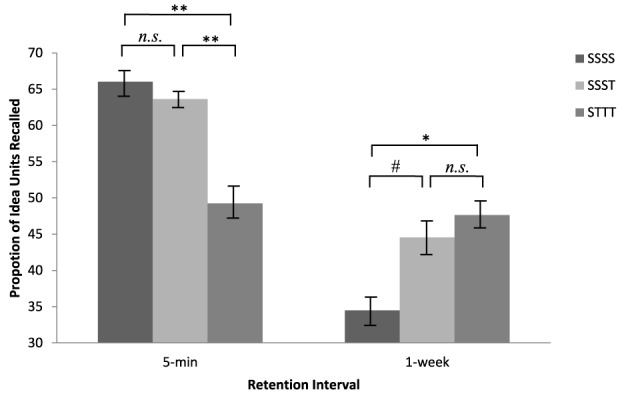
**Mean proportion of idea units recalled during the final free recall test, as a function of learning conditions (SSSS, SSST, or STTT) and learning intervals (5-min or 1-week)**. S denotes study; T denotes test. Error bars represent standard errors. ^*^Denotes difference is significant at *p* < 0.05, ^**^denotes difference is significant at *p* < 0.01, ^#^denotes difference is marginally significant at *p* = 0.07.

A 3 × 2 × 2 between-subjects analysis of variance (ANOVA) was performed, with learning condition (SSSS, SSST, or STTT) and retention interval (5-min or 1-week) as the independent variables of primary interest, and video (“Music History” or “Brain Matter”) as the independent variable for control purposes, i.e., to insure that effects, if any, persisted across video types. A graphical representation of the data appears in Figure 1. The three-way interaction did not reach significance, *F* < 1. Importantly, a significant learning condition × retention interval interaction obtained, *F*_(2, 111)_ = 9.88, *MSe* = 21.26, *p* < 0.001, η^2^_*p*_ = 0.15.

To illuminate the specific pattern of results, post hoc analyses were performed. For the 5-min retention interval, participants in the SSSS condition recalled significantly more idea units (*M* = 19.81, *SD* = 4.26) than did participants in the STTT condition (*M* = 14.77, *SD* = 5.31), *t*_(41)_ = 3.42, *p* < 0.005, *d* = 1.06, albeit not significantly more than did participants in the SSST condition (*M* = 19.09, *SD* = 2.89), *t*_(35.02)_ = 0.645, *p* = 0.52. Additionally, participants in the SSST condition recalled more than did the participants in the STTT condition, *t*_(32.42)_ = 3.35, *p* < 0.005, *d* = 1.18. A contrasting trend was observed at the 1-week retention interval: Participants in the STTT condition recalled more idea units (*M* = 14.29, *SD* = 4.49) than did the participants in the SSSS condition (*M* = 10.35, *SD* = 4.66), *t*_(37)_ = 2.69, *p* < 0.05, *d* = 0.88, albeit not significantly more than did the participants in the SSST condition (*M* = 13.37, *SD* = 5.51), *t*_(36)_ = 0.57, *p* = 0.57. Additionally, participants in the SSST condition recalled marginally significantly more than did the participants in the SSSS condition, *t*_(37)_ = 1.85, *p* = 0.07, *d* = 0.61. These data, taken together, suggest that repeated studying is useful only for short-term retention of video-based knowledge whereas testing inoculates that knowledge for a longer time.

Finally, we measured—in addition to mean proportions of idea units recalled on the final free recall test—mean rates of forgetting over time, using a proportional measure:

$$\begin{array}{l} \frac{\left(initial\;proportion\;of\;recall\right)\;-\;\left(final\;proportion\;of\;recall\right)}{\left(initial\;proportion\;of\;recall\right)} \end{array}$$

**Figure 2:**
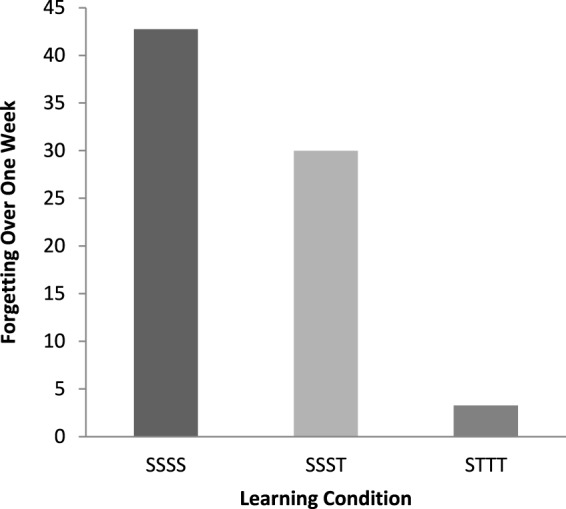
**Proportional measures of forgetting over a week as a function of learning conditions (SSSS, SSST, or STTT)**. S denotes study; T denotes test.

The proportional measures of forgetting are displayed in Figure 2. Participants in the SSSS condition (42.75%) forgot far more than did participants in the SSST condition (29.97%) or the STTT condition (3.25%). The proportional-forgetting analyses suggest that testing reduced occurrences of forgetting (see, also, Wheeler and Roediger, 1992).

## Discussion

The data supported our predictions. Participants who studied the *Coursera* video-based materials repeatedly performed better than did those who tested themselves repeatedly, when the final test was administered immediately. In contrast, participants who studied repeatedly were in fact outperformed by participants who tested themselves, when the final test was administered a week later. That testing enhanced longer-term retention of video-based knowledge is compatible with previous findings documented in the testing effect literature. Researchers have now sought to extend research on testing to meaningful learning—the learning of complex educational materials involving inference making and knowledge application (see Karpicke and Grimaldi, 2012, for an excellent review).

Our data are intended to motivate future studies in extending the validity of research from such controlled contexts as ours in this study to naturalistic online learning environments. We have, for instance, begun investigating in our Lab how mnemonic benefits of testing-based learning involving instructional videos can be translated to ecologically relevant educational contexts—designed into easy-to-implement educational interventions—to aid learners who are embarked on actual *Coursera* courses. We endeavor to delineate the necessary conditions for real-world educational interventions to thrive and, ultimately, help learners discover the strategies they need to support their own educational goals.

## Author contributions

PY designed the study, collected and analyzed the data, and wrote the manuscript; SL designed the study, analyzed the data, and wrote the manuscript.

### Conflict of interest statement

The authors declare that the research was conducted in the absence of any commercial or financial relationships that could be construed as a potential conflict of interest. The reviewer, Dalya Samur, and handling Editor declared their shared affiliation, and the handling Editor states that the process nevertheless met the standards of a fair and objective review.

## References

[B1] Balota D. A. Duchek J. M. Paullin R. (1989). Age-related differences in the impact of spacing, lag, and retention interval. Psychol. Aging 4, 3–9. 10.1037/0882-7974.4.1.3 2803609

[B2] Bangert-Drowns R. L. Kulik J. A. Kulik C. C. (1991). Effects of frequent classroom testing. J. Educ. Res. 85, 89–99. 10.1080/00220671.1991.10702818

[B3] Bohannon J. (2015). Many psychology papers fail replication test. Science 349, 910–911. 10.1126/science.349.6251.910 26315412

[B4] Butler A. C. Roediger H. L. (2007). Testing improves long-term retention in a simulated classroom setting. Euro. J. Cogn. Psychol. 19, 514–527. 10.1080/09541440701326097

[B5] Carrier M. Pashler H. (1992). The influence of retrieval on retention. Mem. Cogn. 20, 633–642. 10.3758/BF03202713 1435266

[B6] Chan J. C. K. McDermott K. B. Roediger H. L. III. (2006). Retrieval induced facilitation: initially nontested material can benefit from prior testing. J. Exp. Psychol. Gen. 135, 553–571. 10.1037/0096-3445.135.4.553 17087573

[B7] Johnson C. I. Mayer R. E. (2009). A testing effect with multimedia learning. J. Educ. Psychol. 101, 621–629. 10.1037/a0015183

[B8] Karpicke J. D. (2012). Retrieval-based learning: active retrieval promotes meaningful learning. Curr. Dir. Psychol. Sci. 21, 157–163. 10.1177/0963721412443552

[B9] Karpicke J. D. Grimaldi P. J. (2012). Retrieval-based learning: a perspective for enhancing meaningful learning. Educ. Psychol. Rev. 24, 401–418. 10.1007/s10648-012-9202-2

[B10] Leeming F. C. (2002). The exam-a-day procedure improves performance in psychology classes. Teach. Psychol. 29, 210–212. 10.1207/S15328023TOP2903_06

[B11] Lim S. W. H. Ng G. J. P. Wong G. Q. H. (2015). Learning psychological research and statistical concepts using retrieval-based practice. Front. Psychol. 6:1484. 10.3389/fpsyg.2015.01484 26500573 PMC4593518

[B12] Open Science Collaboration (2015). Estimating the reproducibility of psychological science. Science 349:aac4716. 10.1126/science.aac4716 26315443

[B13] Pappano L. (2012, November 2). The Year of the MOOC. The New York Times. Available online at: http://www.nytimes.com/2012/11/04/education/edlife/massive-open-online-courses-are-multiplying-at-a-rapid-pace.html.

[B14] Pyc M. A. Rawson K. A. (2010). Why testing improves memory: mediator effectiveness hypothesis. Science 330, 335. 10.1126/science.1191465 20947756

[B15] Roediger H. L. Karpicke J. D. (2006a). Test-enhanced learning: taking memory tests improves long-term retention. Psychol. Sci. 17, 249–255. 10.1111/j.1467-9280.2006.01693.x 16507066

[B16] Roediger H. L. III Karpicke J. D. (2006b). The power of testing memory: basic research and implications for educational practice. Perspect. Psychol. Sci. 1, 181–210. 10.1111/j.1745-6916.2006.00012.x 26151629

[B17] Schacter D. L. Szpunar K. K. (2015). Enhancing attention and memory during video-recorded lectures. Scholar. Teach. Learn. Psychol. 1, 60–71. 10.1037/stl0000011

[B18] Szpunar K. K. Jing H. G. Schacter D. L. (2014). Overcoming overconfidence in learning from video-recorded lectures: implications of interpolated testing for online education. J. Appl. Res. Mem. Cogn. 312, 161–164. 10.1016/j.jarmac.2014.02.001

[B19] Szpunar K. K. Khan N. Y. Schacter D. L. (2013). Interpolated memory tests reduce mind wandering and improve learning of online lectures. Proc. Natl. Acad. Sci. U.S.A. 110, 6313–6317. 10.1073/pnas.1221764110 23576743 PMC3631699

[B20] Wheeler M. A. Roediger H. L. III. (1992). Disparate effects of repeated testing: reconciling Ballard's (1913) and Bartlett's (1932) results. Psychol. Sci. 3, 240–245. 10.1111/j.1467-9280.1992.tb00036.x

[B21] Wittmaier B. C. (1972). Test anxiety and study habits. J. Educ. Res. 65, 352–354. 10.1080/00220671.1972.10884344

