# Supplementary material for: Observing the Testing Effect using Coursera Video-Recorded Lectures: A Preliminary Study
Source: Front Psychol. 2016 Jan 29;6:2064. doi: 10.3389/fpsyg.2015.02064 (PMC4731506; doi:10.3389/fpsyg.2015.02064)
Supplement: Supplementary file 1 [file DataSheet1.docx]

**Supplementary Material**

**Video lecture transcript excerpts (verbatim) reflecting idea units:**

“Music History”

This week, we'll turn our attention to the _(1)_ world of popular music in America before _(2)_ Rock and Roll really broke in 1955… most scholars will say, that _(3)_ Rock and Roll, was the, the *[sic]* result of blending together of three styles that had been prominent in popular music up to 1955. And those styles are _(4)_ mainstream Pop, _(5)_ Country and Western music…

“Brain Matter”

_(1)_ Brain weighs about three pounds which _(2)_ is about 2% of our total body weight… _(3)_ We have the biggest brain of any animal relative to the size of our body… So, _(4)_ at 2%, we have the largest brain to body ratio. But the really interesting thing is the brain is only 2% of the body, but look at the resources it consumes. _(5)_ 25% of our oxygen, so one of every four breaths you take goes to your brain...
